# Ultrasound-Guided Sialendoscopy Surgery for Parotid Sialolithiasis Using Yttrium Aluminum Garnet (YAG)-Holmium Laser: A Prospective Case Series

**DOI:** 10.7759/cureus.65790

**Published:** 2024-07-30

**Authors:** Hong Loi Nguyen, Xuan Phu Tran, Kim Tuan Nguyen, Van Khanh Nguyen, Quang Hien Nguyen Viet, Nguyen Huu Son, Anh Tuan Nguyen, Tien Duc Nguyen Van, Dinh Hoa Nguyen, Nhu Hiep Pham

**Affiliations:** 1 Odonto-Stomatology Center, Hue Central Hospital, Hue, VNM; 2 Urology Department, Hue Central Hospital, Hue, VNM; 3 Anesthesia and Resuscitation Department, Hue Central Hospital, Hue, VNM; 4 Pediatric Center, Hue Central Hospital, Hue, VNM; 5 Radiology Department, Hue Central Hospital, Hue, VNM; 6 Ultrasound Department, Hue Central Hospital, Hue, VNM; 7 Pediatric and Acute Abdominal Surgery Department, Hue Central Hospital, Hue, VNM

**Keywords:** outcomes, parotid glands, holmium yag laser, salivary gland stones, endoscopic surgery

## Abstract

Background: Parotid sialolithiasis is a common condition in middle-aged individuals, with most cases occurring in the submandibular and sublingual glands, followed by the parotid glands and minor salivary glands. The treatment of salivary gland stones, particularly those of the parotid glands, remains challenging. Endoscopic surgery using a yttrium aluminum garnet (YAG)-holmium laser for parotid sialolithiasis is a minimally invasive approach that provides effective treatment for patients. This study aimed to evaluate the outcomes of the endoscopic laser treatment of parotid sialolithiasis a YAG-holmium laser.

Materials and methods: A prospective case series study was conducted on 21 patients diagnosed with salivary gland stones in the parotid gland based on clinical features and imaging findings (including ultrasound and computed tomography scans), from March 2022 to March 2024. These patients underwent sialendoscopy surgery using a YAG-holmium laser and were evaluated for surgical results at 2, 4, and 12 weeks.

Results: Cases with completely reduced symptoms accounted for 90.5%, whereas cases with partially reduced symptoms accounted for 9.5%. The ultrasound image of the salivary gland after surgery was significantly improved compared to that before surgery. After three months of surgery, most patients (90.5%) were satisfied. The postoperative complication rate was 14.3%, which included scarring at the opening of the salivary gland and in the salivary duct.

Conclusion: Sialendoscopic surgery using a YAG-holmium laser for parotid sialolithiasis is a minimally invasive surgical intervention that leaves no scarring, reduces the risk of complications as seen in open surgery, and shortens the postoperative care time for patients.

## Introduction

Parotid sialolithiasis is defined as calcium stones located within the ducts or glandular tissues of the salivary glands [1]. Calculi are mainly found in the submandibular glands (80-85%), followed by the parotid glands (5-10%), and account for 5% of the sublingual glands [2]. The most common clinical symptoms are swelling and pain in at salivary gland area post meals. Subsequently, symptoms, such as localized inflammation, may cause pain and limited mouth opening. In more severe cases such as cellulitis, gland fibrosis, or fistula formation it can occur if the salivary gland calculi are not treated [3,4]. The current standard of care for salivary gland stones includes conservative management such as hydration, sialogogues, and massage. In cases where these measures fail, surgical interventions like sialendoscopy, extracorporeal shock wave lithotripsy, or open gland excision are considered. Sialendoscopy is increasingly preferred due to its minimally invasive nature and ability to preserve gland function. The most common treatment method is complete removal of the affected salivary gland containing stones. In some cases, surgical removal of stones in the mouth is indicated when the stones are solitary and palpable [5]. Endoscopic laser lithotripsy, initially used for breaking urinary stones, has been applied in the treatment of salivary gland calculi, showing promising results in initial studies [6,7]. The first report of laser application in treating salivary gland calculi by Gundlach et al. (1990) documented a 92% success rate for stone clearance using an excimer laser [8]. Subsequently, Marchal et al. reported that holmium and erbium lasers improved the success rate of complex stone treatment from 35% to 70% [9]. Thereafter, many laser systems were developed using gases (e.g., excimers), liquids (e.g., dyes), or solid substances (e.g., neodymium:YAG {yttrium aluminum garnet}, holmium:YAG, erbium:YAG, and thulium:YAG).

However, few studies have been conducted on these systems. Recent studies have reported success rates above 80%, mostly using YAG-holmium lasers [5,10]. It is unclear whether the composition of stone components can affect surgical outcomes, but some in vitro studies have found that YAG-holmium lasers are effective in breaking stones regardless of their physical or optical properties. Combined with recent clinical study results and considering the cost-effectiveness of the method, the YAG-holmium laser remains a suitable choice for laser endoscopic surgery [5,11]. The potential advantages of using a YAG-holmium laser in endoscopic treatment include precise targeting of stones with minimal damage to surrounding tissues, reduced risk of complications, and faster recovery times compared to open surgery. The YAG-holmium laser's high-energy pulses effectively fragment stones, facilitating their removal and alleviating obstruction. The risks of endoscopic surgery using lasers include thermal damage to the surrounding soft tissue, blood vessels, or nerves and perforation of the gland duct wall, which may occur in approximately 13% of cases. This can be avoided by careful irrigation, which also facilitates easier stone removal. However, excessive irrigation can lead to complications such as mouth floor swelling or glandular tissue edema. Therefore, we conducted this study to evaluate the outcomes of endoscopic laser treatment of parotid sialolithiasis using a YAG-holmium laser.

## Materials and methods

Study population

A prospective descriptive study was conducted on 21 patients diagnosed with parotid gland sialolithiasis at the Hue Central Hospital, Hue, Vietnam. The patients underwent endoscopic surgery using a YAG-holmium laser, from March 2022 to March 2024. The institutional review board of Hue Central Hospital issued approval 653/QD-BVH. This case series has been reported in accordance with the PROCESS (Preferred Reporting of Case Series in Surgery) guidelines [12].

The inclusion criteria were (1) patients with recurrent swelling and pain in the parotid gland post meals, and parotid sialadenitis due to failed internal medicine treatment; (2) ultrasonography revealing strong echogenic bands or distant hypoechoic shadows; (3) dilated ducts clearly observed in the cases of ductal obstruction; and (4) computed tomography (CT) demonstrating radiopaque stone structures in the parotid glands.

The exclusion criteria were: (1) acute suppurative parotid sialadenitis, severe stenosis, and adhesions due to previous oral surgery; (2) other lesions causing bleeding and making intervention difficult; (3) contraindications for surgery due to internal and external diseases, anesthesia; and (4) inability to agree to participate in the study, insufficient study records, inability to follow-up, and lack of follow-up appointments.

Surgical techniques

To perform the surgical procedure, we used these pieces of equipment: Themis Laser Lithotripter (Initia, Israel); 4D Color Doppler Ultrasound System Model Prosound 7 (Hitachi Aloka Medical Ltd, Japan); endoscopic system including light source, camera, monitor, 1.3 mm diameter endoscope; fiber optic cable; guidewire; oral surgical instruments: molt mouth prop, needle holder, forceps, surgical suction machine, and loupe glasses.

Before surgery, all patients underwent ultrasound and CT scans of the parotid gland area, pre-operative examination, surgical planning, preoperative treatment of the oral cavity, and ultrasound examination of the stone location.

Intraoperative identification of Stensen's duct openings inside the mouth and face was conducted. The catheter and endoscopic guidewire were placed through the catheter under ultrasound guidance to reach the location of the stone. Fixation of endoscopic guidewire and catheter removal was performed by passage of 4 Fr, 6 Fr, 8 Fr, and 10 Fr dilators through the guidewire under ultrasound guidance, reaching the submandibular stone. The lithotripter parameters were checked and the endoscope was positioned to approach the stone under ultrasound guidance. Fragmentation of stones using a YAG-holmium laser was performed with a power setting of 6 W, frequency of 10 Hz, and energy level of 0.6 J. A flexible catheter (6-8Fr) was inserted through the guidewire under ultrasound guidance. Next, the guidewire was removed, and the flexible catheter was sutured to the mucosa near the Stensen's duct opening and lip mucosa.

Postoperatively, antibiotics, anti-inflammatory drugs, and pain-relieving medications were administered, along with massage and warm compression of the glands. Irrigation with normal saline (NaCl 0.9%) was performed using the flexible catheter and the flexible catheter was retained for four to six weeks.

Follow-up

All patients were evaluated for surgical results at 2, 4, and 12 weeks using clinical and imaging evaluations (ultrasound and endoscopy). The results were categorized based on the following criteria: "good" if all evaluation criteria were satisfactory; "average" if one evaluation criterion was rated as "average;" and "poor" when one evaluation criterion was rated as poor.

Statistical analysis

Statistical analyses were performed using IBM SPSS Statistics for Windows, version 22.0 (IBM Corp., Armonk, NY, USA). Continuous variables were presented as means, medians, and ranges. Categorical variables were presented as numbers and percentages.

## Results

Among the 21 subjects included in the analysis, there were 13 males and eight females, with a mean age of 45.2 years (range = 30-65 years). The main symptoms, as well as the reasons for hospitalization, are listed in Table 1. Most patients were examined for symptoms of swelling and pain in the salivary gland area (47.6% and 23.8%, respectively), followed by pus discharge through the salivary duct opening (19%), and dry mouth (9.8%). The majority of symptoms (90.5%) seemed to occur post-meals.

**Table 1:** Symptoms of parotid sialolithiasis

Symptoms	n (%)
Swelling	10 (47.6)
Pain	5 (23.8)
Pus discharge	4 (19.0)
Dry mouth	2 (9.8)
Symptoms related to meals	19 (90.5)

Table 2 presents the clinical features of the patients. Palpable stones in the mouth were found in 19.1% of cases. Furthermore, 90.5% of cases exhibited easily identifiable salivary duct openings.

**Table 2:** Clinical examination

Clinical examination		n (%)
Palpable stones		4 (19.1)
Able to identify salivary duct opening	Easy to identify	19 (90.5)
	Difficult to identify	2 (9.5%)

**Figure 1:**
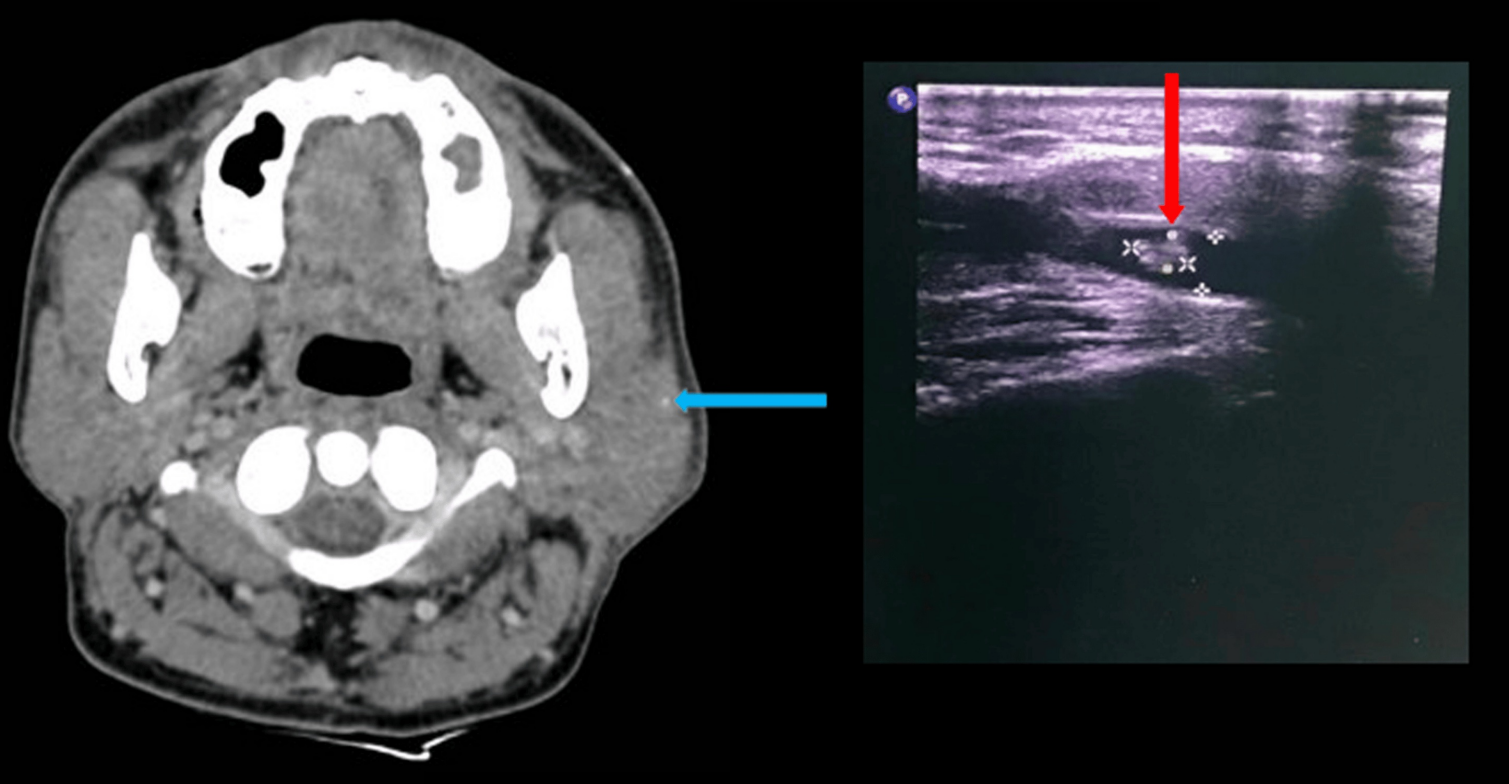
Ultrasound shows the left parotid salivary gland with a dilated duct (red arrow), and CT scan reveals the stone as a well-defined border area (green arrow)

The location of salivary gland lesions detected by ultrasound was most common in the umbilical gland (42.9%), followed by the minor duct (28.6%) (Table 3). CT scans and ultrasound of the parotid gland area are shown in Figure 1.

**Table 3:** Location of stones detected by ultrasound

Location of stones	n (%)
Main duct	4 (19.0)
Umble gland	9 (42.9)
Minor duct	6 (28.6)
Not detected	2 (9.5)

The location of salivary gland stones detected by CT was most common in the parotid gland (47.6%), followed by the main duct (33.3%). The location of salivary stones detected by endoscopy was most common in the umbilical gland (47.6%), followed by the main duct (38.1%) (Table 4). 

**Table 4:** Location of salivary gland stones detected by CT scan and endoscopy

Location on CT	n (%)
Main duct	7 (33.3)
Umble gland	10 (47.6)
Minor duct	3 (14.3)
Not detected	1 (4.8)
Location detected by endoscopy	n (%)
Main duct	8 (38.1)
Parotid gland	10 (47.6)
Parotid duct	3 (14.3)

All patients underwent successful surgery with the average operation time being 45 minutes (range: 30-60 minutes). At three months after surgery, approximately 90.5% of cases had completely reduced symptoms, whereas 9.5% of cases had partially reduced symptoms (Table 5).

**Table 5:** Clinical symptoms at three months after surgery

Degree of improvement	n (%)
Completely reduced	19 (90.5)
Partially reduced	2 (9.5)
No improvement	0 (0)

The ultrasound findings of the salivary gland significantly improved after surgery compared to that before surgery, with a statistically significant difference (p <0.05) (Table 6).

**Table 6:** Endoscopic ultrasound findings at three months after surgery

Endoscopic ultrasound	n (%)
Image of stones	1 (4.7)
Salivary duct obstruction	1 (4.7)
Normal	19 (90.6)
Total	21

Three months after surgery, 90.5% of patients reported satisfaction with the outcomes (Table 7).

**Table 7:** Short-term outcomes (at three months after surgery)

Results	n (%)
Good	19 (90.5)
Average	2 (9.5)
Poor	0 (0)

The postoperative complication rate was 3/21 (14.3%), including scars at the opening of the salivary gland and scars in the salivary duct (Table 8).

**Table 8:** Post-operative complications

Complication	n (%)
Scar at the opening of the salivary gland	2 (9.5)
Scar in the salivary duct	1 (4.8)
Total	3 (14.3)

## Discussion

Clinical characteristics

Regarding reasons for hospitalization, common complaints in the study were swelling and pain on one side of the submandibular or parotid gland, occurring repeatedly at rates of 45.7% and 25%, respectively. Additionally, some patients presented with pus discharge from the opening of the salivary gland and dry mouth. According to the studies by Koch et al. and Pachisia et al., 12-18% of patients presented with swelling and pain in the salivary gland area, accompanied by pus discharge at the opening of the salivary gland [13,14]. The clinical symptoms in patients may include one or more combined symptoms of salivary gland stone diseases, such as swelling, pain, pus discharge, and dry mouth. Most patients presented with swelling, pain in the salivary gland area, and pus discharge through the opening of the salivary gland at rates of 47.6%, 23.8%, and 19%, respectively. We believe the symptoms of swelling and pain in the parotid gland area are caused by these diseases, causing obstruction of the salivary duct and leading to salivary stasis and increased pressure in the salivary duct. In cases where patients do not have symptoms of swelling and pain because the salivary duct is not completely obstructed, saliva can still seep through the gaps without being stagnant in the salivary duct [13]. Of these patients, 90.5% noted symptoms related to meals, particularly those that worsened after meals. During clinical examination, no cases of swelling or pain in the salivary gland area accompanied by signs of infection, such as heat, redness, or pus discharge from the gland opening, were recorded. This absence may be attributed to prior internal treatment in most patients. In addition, patients were evaluated for abnormalities in the facial jaw area, such as asymmetrical chin, protruding teeth, and reverse growth. The location of the opening of the parotid gland was initially determined to predict the difficulty level in the salivary gland endoscopic technique.

Imaging characteristics (ultrasound, CT scan) and salivary gland endoscopy

Ultrasound provides an overview of the glandular ducts. Ductal injuries on ultrasound are divided into two main groups: stone-related ductal obstruction and non-stone-related ductal obstruction, with stone-related obstruction accounting for the majority of cases. Ultrasound can show shadow images of stones or indirectly show obstruction due to dilation of the ductal system behind the site of the obstruction. In this study, the site of injury on ultrasound was mainly the renal pelvis, followed by the ductal tissue. Additionally, ultrasound can diagnose some characteristics of stone-related injuries, such as the number and size of stones. According to a 2017 study by Thomas et al. on the accuracy of ultrasound and CT scans compared to endoscopic retrograde cholangiopancreatography in biliary stone disease [15], the authors found that ultrasound had many advantages in diagnosing biliary stone diseases because it is easy to perform, noninvasive, not affected by X-rays, and cost-effective. However, ultrasound relies heavily on physician experience. In some cases, such as stones smaller than 4 mm, stones located in the distal part of the main duct, multiple stones, and stones with low mineral content, ultrasound may not provide a diagnosis, and other imaging modalities, such as CT scans or endoscopic retrograde cholangiopancreatography, may be required.

The classification of ductal damage on a CT scan is the same as that on ultrasound, which is divided into two main groups: stone obstruction and non-stone obstruction, in which stone obstruction accounts for the majority of the cases. In our study, duct damage to the hilum of the gland, the main duct, accounted for the highest proportion. For ductal stone disease, CT scans determine a number of stone pathological characteristics, such as the number of stones, stone size, level of radiopacity, and stone density, thereby helping to diagnose and evaluate the damage. Injury and treatment of the disease. A CT scan can detect multiple stones in the duct that may not be visible on ultrasound. CT scans are also useful for diagnosing pathologies in the glandular parenchyma and surrounding organs that can cause duct blockages. Non-contrast CT scans help diagnose stone diseases, whereas contrast-enhanced CT scans more accurately diagnose cases with complications or other accompanying salivary gland diseases, such as abscesses, cysts, or tumors.

We evaluated several symptoms related to the images through endoscopic examination of the glandular tube, such as the condition of the glandular tube mucosa, circular swelling around the glandular tube, fiber discharge, nodules, and granules. Initially, we noticed changes in the glandular tube system in this pathological group. The glandular tube mucosa is generally smooth and lined with a pale-colored connective tissue layer, with visible blood vessels on the surface of the connective tissue layer and no nodules or granules inside the glandular tube. In addition, circular swelling around the glandular tube was observed owing to the constriction mechanism of the muscle around the glandular tube, which was most clearly visible in the glandular bud area. Similar to the results of our study, Koch et al., Katz et al., and Yu et al. found that fiber discharge and nodules partially or completely obstructed glandular tubes [13,16,17]. We found that the glandular buds of the glandular tube usually have the following characteristics: they are dense, lumpy, and opaque, often blurring the endoscopic image and making it difficult to observe the glandular tube image.

Short-term outcomes

The symptoms showing the greatest improvement post-surgery were fever, changes in taste, and excessive salivation, with a 90.5% improvement rate. This was followed by reductions in pus discharge, pain, and swelling in the salivary gland. Patients with postoperative dry mouth symptoms were included in the salivary gland removal surgery group. Therefore, after endoscopic and combined endoscopic surgeries, clinical symptoms improved significantly, increasing the quality of life of patients [18,19].

The endoscopic images that showed the greatest improvement after surgery were granules and fiber discharge, which improved by 90.5%. Therefore, after endoscopic and combined endoscopic surgery, the endoscopic images improved significantly.

The ultrasound results after surgery showed that the stone images improved by 90.6%. Therefore, after endoscopic and combined endoscopic surgeries, the ultrasound images improved significantly.

Three months after surgery, most patients (90.5%, average 9.5%) were satisfied compared to before surgery, with good results after three months, and most symptoms improved completely. These results demonstrated the effectiveness of sialendoscopy surgery for parotid sialolithiasis using a YAG-holmium laser.

The complication rate after single- or combined-endoscopic interventions was 14.3% (three cases), including narrow scarred duct openings and narrow ducts. Complications such as facial nerve damage, bleeding, surgical site infections, and duct strictures were not recorded in our study. Three cases of narrow scarred duct openings were observed following open duct surgery. In these cases, we could not find the duct during endoscopy; therefore, we actively opened it. Two weeks later, the patient had a narrow scarred duct opening. We reopened the duct, placed a stent at the duct opening for four weeks, and then removed the tube for examination and follow-up without stricture recurrence.

Limitations of the study

The limitations of this study are the small sample size (21 patients), lack of a control group, and short-term follow-up (three months). More studies might be required to confirm the effectiveness of this system. Nevertheless, this is the first study in Vietnam that applied sialendoscopy surgery for parotid sialolithiasis using a YAG-holmium laser. The results of this study provide further evidence for the effective and safe use of YAG-holmium laser among Vietnamese patients.

## Conclusions

By applying the yttrium aluminum garnet (YAG)-holmium laser in parotid gland sialolithiasis endoscopic surgery, we found that this method was minimally invasive; resulted in reduced scarring compared to open surgery; had reduced risk of complications such as bleeding, nerve damage, and facial paralysis; shortened postoperative care time for patients; ultrasound-assisted examination of stone locations, fibrosis, and duct stenosis; accurate determination of the laser contact position with the stone; increased efficiency of stone fragmentation; and evaluation of postoperative follow-up results. The effectiveness of multidisciplinary cooperation was also demonstrated.
